# Health equity profile of knee replacement patients in the South African public sector: A descriptive study

**DOI:** 10.4102/sajp.v80i1.2027

**Published:** 2024-07-18

**Authors:** Marisa Coetzee, Amanda M. Clifford, Jacobus D. Jordaan, Quinette A. Louw

**Affiliations:** 1 Division of Physiotherapy, Health and Rehabilitation Sciences, Faculty of Medicine and Health Sciences, Stellenbosch University, Cape Town, South Africa; 2 Health Research Institute, Ageing Research Centre, School of Allied Health, University of Limerick, Limerick, Ireland; 3 Department of Orthopaedic Surgery, Faculty of Medicine and Health Sciences, Stellenbosch University, Cape Town, South Africa

**Keywords:** health equity, knee replacement, osteoarthritis, quality of life, PROGRESS-Plus

## Abstract

**Background:**

Knee replacement surgery can significantly improve the quality of life of patients with severe knee osteoarthritis. Equitable access to knee replacement surgery is important to ensure that everyone, regardless of their socioeconomic status or geographical location, have fair and timely access.

**Objectives:**

The aim of our study was to (1) describe the health equity profile and quality of life of patients awaiting knee replacement at a single academic hospital in South Africa and to (2) describe the association between these health equity factors and the waiting time.

**Method:**

A cross-sectional survey and retrospective record review of patients awaiting knee replacement was conducted using the PROGRESS-Plus health equity framework. Chi-square statistics were used to calculate association between health equity factors and the waiting time.

**Results:**

Three-hundred and two (*N* = 302) patients (77% female; mean age 67 years) participated, of whom one in three patients waited 5 years or longer for surgery. Elderly patients (> 70 years) and patients from lower socio-economic background were less likely to have equitable access to surgery.

**Conclusion:**

The current screening protocol for knee replacement surgery in the public health care sector does not provide equitable access to surgery. A more holistic screening approach alongside selective surgical prioritisation and rehabilitation could reduce the waiting list and facilitate equitable access to care.

**Clinical implications:**

Health equity factors such as socioeconomic status, age, and other patient characteristics such as life roles and employability should be taken into consideration when screening patients for elective knee replacement waiting lists.

## Introduction

Osteoarthritis (OA) is a degenerative condition affecting weight-bearing joints such as the knee. As the disease progresses, it leads to joint deterioration and severe pain, causing functional disability (Coaccioli et al. 2022), and in the case of severe OA, joint replacement surgery may be performed (Lewis et al. 2020; Nelson et al. 2014). Joint replacement surgery can significantly improve the quality of life of patients with severe knee OA by reducing pain and enabling regaining mobility to engage in daily activities with greater ease.

The global demand for joint replacement surgery is on the rise, which is expected to result in extended waiting times (Jabbal et al. 2024; Shichman, Askew, Habibi et al., 2023). The average waiting time for joint replacement surgery in public healthcare sectors varies by country. Across developed European countries, Australia and the United States, the waiting times for joint replacement vary from 45 to 495 days (Inacio et al. 2017; Pabinger, Lothaller & Geissler 2015) with an average waiting time of 2 years reported among commonwealth countries in the past 6 years, ranging from under 1 to 9 years (Jabbal et al. 2024; Siciliani, Moran & Borowitz 2014; Tsui & Fong 2018). However, it is important to consider that developed countries spend large amounts of their gross domestic product (GDP) on their healthcare and can provide timely access to elective surgery (Tikkanen et al. 2020).

In South Africa, the elective nature of joint replacement surgery proves challenging to prioritise in the public sector, where there is a high prevalence of traumatic injuries and fractures necessitating emergency surgeries (Hardcastle et al. 2016). These challenges were compounded during the coronavirus disease 2019 (COVID-19) pandemic when services had to be stopped for long periods (Nel 2022). Surgeons screen patients requiring joint replacement, placing them on a waiting list according to disease severity and functional disability. Past waiting times have varied from less than 1 to 8 years, based on the surgical prioritisation score developed by Kavalier, Nortje and Dunn (2017), which is used across South Africa’s public sector to determine the urgency and order of joint replacement surgeries. Long waiting times for joint replacement surgery can lead to mental and functional decline before the procedure, negatively impacting post-operative outcomes, patient well-being and healthcare system strain (Akin-Akinyosoye et al. 2018; Bushnell, Ceko & Low 2013; Karayiannis et al. 2023; Lowry et al. 2021; Rice et al. 2019).

Equitable access to joint replacement surgery is of paramount importance to ensure that all patients, regardless of their socioeconomic status or geographical location, have fair and timely access to this life-changing procedure (Devasenapathy et al. 2020). Considering the inequities of the apartheid regime and how it still affects the majority of South Africans today (De Villiers 2021), through the promotion of equitable access, we can address disparities in healthcare and prioritise needs, fostering a more inclusive and just society. Moreover, providing timely access to joint replacement surgery can prevent the exacerbation of patients’ conditions (Lowry et al. 2021), reducing the burden on healthcare facilities, and it can potentially save healthcare costs. Emphasising equitable access to joint replacement surgery underscores the fundamental principle that access to essential medical interventions should be determined by medical need, rather than financial means, creating a more equitable and compassionate healthcare system for all (Judge et al. 2010).

Research has shown that pre-operative rehabilitation strategies such as exercise improved function and reduce pain intensity, resulting in better post-operative outcomes for TKR (Dash et al. 2017; Sharma, Ardebili & Abdulla 2019). This may delay the need for surgery as some patients may no longer require surgery because of improved symptoms (Dell’Isola et al. 2021; Skou et al. 2016). Understanding the characteristics of patients awaiting TKR is essential as it assists to identify subgroups of patients with OA and informs tailored interventions to meet their specific needs (Nelson 2018; Rice et al. 2019). However, in the South African context, there is limited knowledge regarding the profile of patients awaiting TKR. With an anticipated increase in knee OA diagnoses in South Africa (Gouda et al. 2019) and growing surgical waiting lists in the public health care sector, it is crucial to gain an understanding of the characteristics of patients on the waiting list for TKR to identify and address the needs of these patients equitably.

The aim of our study was to (1) describe the health equity profile and quality of life of patients awaiting TKR at Tygerberg Hospital and to (2) describe the association between these health equity factors and the waiting time.

## Research methods and design

### Study design

A cross-sectional survey and record review of patients awaiting TKR surgery was conducted at Tygerberg Hospital, Cape Town.

### Study setting and participants

Tygerberg Hospital (TBH) is the largest tertiary hospital in the Western Cape serving a total of 3.4 million people from a geographical catchment area of 250 km wide (Western Cape 2016). Patients who receive tertiary health care services at TBH come from various cultural backgrounds, typically speak one of three languages (Afrikaans, English or isiXhosa) and live in either rural, peri-urban or urban settings (Western Cape Government 2013). Patients who are dependent on public health care services typically fall within a lower socio-economic bracket, with their immediate environmental and social circumstances varying. In some areas, patients live in informal settlements with poor sanitation but are closer to the city for employment, frequent public transport opportunities and regular access to rehabilitation services (StatsSA 2020). In comparison, patients from peri-urban and rural settlements often live in brick houses but have restricted opportunity for employment, pay exorbitant fees for transport to travel to the city in order to access tertiary government services and often have sporadic or no access to rehabilitation services (Sherry 2015; Vergunst et al. 2015).

Patients on the TKR waiting list at TBH have undergone a twofold evaluation process to determine their eligibility for surgery. At first, a referral from a primary health care physician is made via an electronic system and typically includes a clinical description with details on the medical history, comorbidities, previous interventions and a recent x-ray. These referrals are screened by the head of the Arthroplasty Clinic and an appointment is scheduled at the clinic. During consultation, a surgical prioritisation score is completed (see Online Appendix 1), and patients are placed on the waiting list according to their score. Typically, the highest scored patients receive surgery first.

### Data collection

A survey was developed and administered by the authors, guided by the PROGRESS-Plus health equity framework (Kavanagh, Oliver & Lorenc 2008), which describes variables affecting equitable access to healthcare. The framework includes place of residence, race, occupation, gender, religion, education, socioeconomic status, social capital, age and disability. The EuroQoL EQ-5D-5L (Feng et al. 2021) was used to collect information on quality of life and additional questions such as the level of education (school grade successfully completed), employment history (currently employed, not employed or pensioner) and employment type during the survey. The electronic clinical records were accessed by the primary author and information on the household income (categorised at state level from H0-H4, see Table 1), home language, area of residence (suburb or town), surgery waiting time (days), date of birth (age) and sex were extracted. All the information was captured onto a Microsoft Excel spreadsheet by the primary author. Only data related to the health equity factors are presented in this article. Data related to clinical presentation will be presented in a separate article. The EuroQoL EQ-5D-5L has been validated in South Africa for all the three major languages spoken in the Western Cape (English, Afrikaans and isiXhosa) as well as for administration over the telephone. It has also been previously used on patients with knee OA (Vitaloni et al. 2019). This questionnaire provides a short descriptive profile and a single summary index value for health status in patients. The descriptive section of the questionnaire consists of five questions measuring five domains (mobility, self-care, usual activities, pain or discomfort and anxiety and/or depression) with a five-level score (between no problem, some problem and extreme problem). Each level is scored from least (1) to worst (5) and the scores of the dimensions provide the index score (e.g. 11 215). The index score was calculated using the crosswalk index value calculator (mapping the EQ-5D-5L data onto the EQ-5D-3L value sets) (Van Hout et al. 2012). Value sets from Zimbabwe were used as no value sets for South Africa were available. The highest value is one (full health) and zero the lowest (as bad as being dead), with values less than zero representing health states worse than death.

**TABLE 1:** Demographic profile of *N* = 302 patients awaiting total knee replacement surgery at Tygerberg Hospital.

Variable	*N*	% of total
**Age group (years)**		
40–49	6	2.0
50–59	52	17.2
60–69	117	38.7
70–79	97	32.1
80–89	29	9.6
90 and above	1	0.3
**Sex**		
Female	235	77.8
Male	67	22.2
**First language**		
Afrikaans	204	67.5
English	53	17.5
isiXhosa	44	14.6
Missing data	1	0.3
**Area of residence**		
Metropole (urban)	278	92.1
Peri-urban	24	7.9
Rural	0	0.0
**Highest level of education**		
No formal education	3	1.0
Primary (Grade 1–7)	56	18.5
Secondary partial (Grade 8–11)	151	50.1
Secondary completed (Grade 12)	65	21.5
Tertiary	20	6.6
Missing data	7	2.3
**Household income†**		
H0 (fully subsidised)	170	56.3
H1 (partially subsidised)	86	28.5
H2 (partially subsidised)	17	5.6
H3 (partially subsidised)	5	1.7
Private (no subsidy)	24	7.9
**Employment**		
Pensioners (total across all income groups)	191	63.2
Economically active age (age under 65)‡	108	35.8
Employed currently (of < 65 year age group)	15	13.9
Unemployed because of knee OA (of < 65 year age group)	51	47.2
Disability Grant (of < 65 year age group)	30	27.8
**Employment type (current or previous)**		
Manual labour	241	79.8
Non-manual labour	61	20.2

OA, Osteoarthritis.

† , Household income: H0 Pensioners and the unemployed (fully subsidised) or SASSA or disability grant; H1 Less than R70 000 single income or R100 000 family income per year (partially subsidised); H2 From R70 000 to R100 000 single income or R250 000 to R350 000 family income per year (partially subsidised); H3 More than R250 000 single income or R350 000 family income per year (partially subsidised); Private (no subsidy).

‡ , Retirement age is typically between 60–65 years in South Africa.

No categorisation tools for employment type (sedentary, light, medium, heavy or very heavy) could be found for data that were already captured; therefore, the authors categorised the data according to manual and non-manual labour based on percentage of the day (> 50%) spent in standing, walking or stair climbing activities, which may affect the load placed on the knee joint.

### Data analysis

For data analysis, the IBM SPSS version 27 was used with 95% confident intervals where appropriate. Frequency distribution (percentages and counts) was used for reporting categorical data (i.e. sex, level of education, income, language, area of residence, employment history and type of employment); means and standard deviation were used for reporting normally distributed continuous variables (i.e. age and waiting time) and median was reported for skewed data. Based on the spread and skewness of the waiting time data, we categorised the data into three groups, namely waiting less than 2 years, waiting between 2 and 5 years and waiting longer than 5 years. Chi-square statistics with a confidence level of 95% were used for exploring the association between categorical waiting time and health equity factors (i.e., age, sex, education level, employment and socioeconomic status) and a post-hoc Bonferroni test was performed to correct for the multiple comparisons between the categories.

### Ethical considerations

Ethical approval to conduct the study was obtained from the Human Research Ethics Committee at Stellenbosch University in March 2021 (reference number S20/11/315 [PhD]). Permission to conduct the study at Tygerberg Hospital was obtained from the manager of medical services through the National Research Database of the Department of Health on 10 May 2021, with extension granted on 08 March 2023. We invited all patients on the waiting list for TKR surgery during the study period to take part in this research. The list, which is continuously updated, was obtained from the provincial administration system at monthly intervals. During the study period, the waiting list had 608 patients, where of 304 patients gave informed consent to participate in the study. Patients were recruited either face-to-face at the arthroplasty clinic or contacted telephonically by a member of staff from the clinic (acting as a research assistant) to introduce them to the project and obtain their informed consent to be contacted by the researcher. Informed consent was obtained from participants in their language of choice (Afrikaans, English or isiXhosa) using a scripted protocol to ensure that information was conveyed in the same way each time. Data were collected from 20 May 2021 until 13 August 2021, after which the record review and data capturing was conducted and completed by 30 November 2021. All data were anonymised, providing each participant a unique identifier code, and data were stored on password-protected devices to ensure the privacy of the participants.

## Results

**FIGURE 1:**
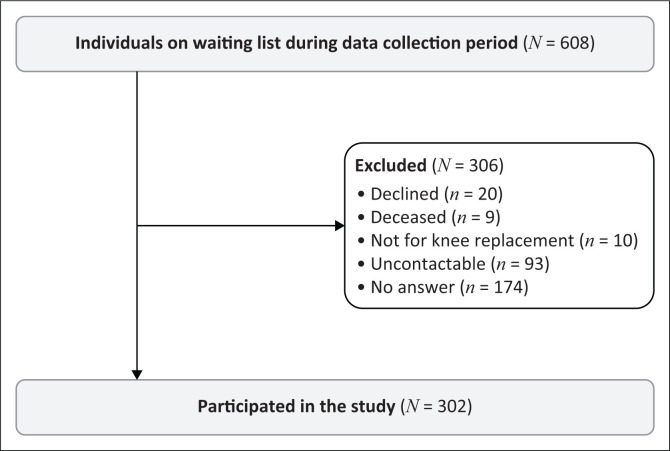
An illustration of the make-up of the patients that participated in the study.

The number of patients contacted are displayed in Figure 1. In addition, 3 patients no longer wished to have surgery (and declined to take part in the study) and 6 patients received surgery either at another public hospital or paid the fees to receive surgery at a private hospital (and were not included in the final total of patients on the waiting list). In comparison, the patients who declined to take part or were uncontactable were similar in age, waiting time and sex, and therefore the patients included were a true representation of the population.

### Health equity profile

The sample consisted of patients with a mean age of 67.5 (s.d. 9) years, and the majority were female (*n* = 235; 77.8%). Over half (*n* = 170; 56.3%) of participants were fully subsidised by the public healthcare system (pensioner, disability grant holder or unemployed) and lived within the metropole of the City of Cape Town (*n* = 278; 92.1%); see Table 1. None of the participants were from the rural areas of the Western Cape.

### Surgery waiting time in relation to equity factors

Participants were waiting a median of 1133 days (IR 608 to 2070.5 days) for surgery with four outlier patients waiting 4217, 4301, 4493 and 6783 days, respectively. Based on the three categories of waiting time, every third person who participated have been waiting 5 years or longer for their surgery at Tygerberg Hospital (Table 2).

**TABLE 2:** Categorised waiting time for surgery for *N* = 301 participants reported as *n* (%).

Time waiting (years)	*n*	% of total
< 2	95	31.6
2–5	103	34.2
> 5	103	34.2

Considering the PROGRESS-Plus health equity factors, less than a quarter (23%) of patients were over 70 years old when added to the list. However, most patients waiting longer than 5 years for surgery were older than 70 years and were likely put on the list at a younger age. There was a significant difference (*p* < 0.0001) in waiting time between the younger and the older age groups. In addition, participants that were not fully subsidised (who must pay a percentage of their hospital costs) were significantly less likely to wait long for surgery (*p* = 0.038) (Table 3).

**TABLE 3:** Categorised waiting time for surgery for *N* = 301 participants reported as *n* (%).

Variables	Association between waiting time and equity factors								Pearson chi-square		
	< 2 years		2–5 years		> 5 years		Total (*N* = 301)				
	*n*	%	*n*	%	*n*	%	*n*	%	Chi-square	*df*	Sig. (2 tailed)
**Age (years)**	-	-	-	-	-	-	-	-	22.772	4	< 0.001*
< 59	29	50.9	17	29.8	11	19.3	57	18.9	-	-	-
60–69	41	35.0	42	35.9	34	29.1	117	38.9	-	-	-
> 70	25	19.8	44	34.6	58	45.7	127	42.2	-	-	-
**Age added to the list (years)**	-	-	-	-	-	-	-	-	2.061	4	0.725
< 59	35	33.3	31	29.5	39	37.1	105	34.9	-	-	-
60–69	40	31.7	47	37.3	39	31.0	126	41.7	-	-	-
> 70	20	28.6	25	35.7	25	35.7	70	23.3	-	-	-
**Education**	-	-	-	-	-	-	-	-	0.266	2	0.876
Not completed sec. education	67	30.9	74	34.1	76	35.0	217	72.1	-	-	-
Completed sec. education	28	33.3	29	34.5	27	32.1	84	27.9	-	-	-
**Gender**	-	-	-	-	-	-	-	-	1.802	2	0.406
Female	73	31.3	76	32.6	84	36.1	233	77.4	-	-	-
Male	22	32.4	27	39.7	19	27.9	68	22.6	-	-	-
**Employment**	-	-	-	-	-	-	-	-	3.367	2	0.186
Unemployed because of OA	29	39.7	24	32.9	20	27.4	73	24.3	-	-	-
Unemployed or other	66	29.1	78	34.4	83	36.6	227	75.4	-	-	-
Unemployed because of OA	29	39.7	24	32.9	20	27.4	73	24.3	0.110	2	0.946
Unemployed	8	36.4	8	36.4	6	27.3	22	7.3	-	-	-
**Socioeconomic status**	-	-	-	-	-	-	-	-	6.543	2	0.038*
Fully subsidised	44	25.9	60	35.3	66	38.8	170	56.5	-	-	-
Not fully subs.	51	38.9	43	32.8	37	28.2	131	43.5	-	-	-

OA, Osteoarthritis; *df*, degrees of freedom; sec., secondary; subs., subsidised; Sig. (2-tailed), significance level.

* , significant difference.

### Quality of life

A quality-of-life score was captured for 296 participants (*n* = 6 missing values) and are displayed in Table 4. The mean score was 67.95 (range 10 to 100; s.d. 18.1). More than half the participants reported having severe or extreme mobility challenges (57%) and pain (60.5%). Most participants reported having difficulty performing usual activities (72.8%), and more than half experience anxiety and/or depression (55.6%).

**TABLE 4:** Euro-QOL-5D-5L scores for *N* = 296 participants reported as *n* (%).

Response level	Mobility		Self-care		Usual activities		Pain		Anxiety and/or depression	
	*n*	%	*n*	%	*n*	%	*n*	%	*n*	%
None	20	6.6	164	54.3	82	27.2	12	4.0	134	44.4
Slight	44	14.6	42	13.9	43	14.2	35	11.6	71	23.5
Moderate	61	20.2	56	18.5	79	26.2	67	22.2	51	16.9
Severe	147	48.7	26	8.6	55	18.2	130	43.0	23	7.6
Unable or extreme	25	8.3	9	3.0	37	12.3	53	17.5	17	5.6

A chi-square test showed participants waiting longer for surgery had a statistically significant (< 0.001) chance of experiencing more severe levels of anxiety than those waiting for a shorter time. The index values had a median of 0.6 and interquartile range of 0.46–0.7. Six patients reported their health-related quality of life to be worse than death.

## Discussion

The findings of our study provide insight into selected health equity factors related to the waiting time and quality of life of patients awaiting TKR surgery at one of the largest academic hospitals in South Africa. Health equity factors included were age, sex, level of education, employment history and type, household income, home language and area of residence. These equity factors are becoming increasingly important in health science research and access to care to ensure that everyone, especially vulnerable groups receive equitable access to quality healthcare.

A key finding was that one in three patients on the waiting list has been waiting for longer than 5 years. South Africa has a high incidence of trauma (violence) compared to the rest of the world, which places a burden on the already limited health resources (Zaidi et al. 2019). Countries with a public health care system similar to South Africa have waiting lists for elective surgery to direct already limited resources towards urgent medical care (OECD 2020). In addition, the COVID-19 pandemic has increased the waiting times of elective surgery in South Africa as these services were stopped for months at a time (Nel 2022). Extended waiting times, as evidenced by Soni et al. (2019), adversely affect patients’ pain sensitisation, mental health, quality of life and post-operative outcomes. In countries such as South Africa, with constrained resources and worsening economic climate (Al-Worafi 2023), prudent measures are needed to mitigate the impact of waiting time.

Published approaches to reduce waiting time include the use of prioritisation criteria. These criteria typically include knee range, walking ability, pain, limitation of ‘other’ functional activities, radiographic findings and impact on life roles (Escobar et al. 2007). Based on a combination of these criteria, patients are classified as a high and low priority for surgery. Service re-orientation models, for example, physiotherapy-led clinics, showed that many patients who are classified as low priority can be referred for effective conservative management at primary care level, thereby reducing the number of patients placed on the waiting list (Pike et al. 2021; Samsson, Grimmer, Larsson et al., 2020). Interventions such as education, weight management, exercise and cognitive behavioural therapy have been found to be effective in treating these patients (Foo et al. 2020; Tong et al. 2020). Consequently, waiting lists and waiting time are reduced, thereby facilitating access to care for those who are most in need. In lower resource settings, such service models could also result in notable savings in healthcare costs by reducing the number of surgeries and complications related to long waiting times.

We found that patients older than 70 years have waited longer compared to the younger age groups as patients have aged on the waiting list. This finding is of concern as functional decline is already linked to ageing patients, and in the presence of moderate to severe painful osteoarthritis, it may intensify their functional disability and increase their need for surgery and higher likelihood of a poorer outcome. Furthermore, the overall health risk profile (comorbidities) of the relatively older group most likely also deteriorates over time, and in the presence of disabling pain, they are forced into sedentary lifestyles (Fernandez-Fernandez & Rodriguez-Merchan 2015; Wang et al. 2016). These compounding factors coupled with a long waiting time may reduce their chances of successful surgical outcomes, increase their chances of developing cardiometabolic disease (Wang et al. 2016) and render them more at risk for not receiving surgery because of their health risk profile. In addition, the risk of falling and the negative physical and psychosocial consequences of falls could increase the healthcare need and cost to the person and the healthcare system (Zhang et al. 2023). A review done by Cheng et al. (2015), highlighted the increase in mental distress, loss of independence and reduced health-related quality of life (HRQoL) as an effect of longer waiting times on older adults (Cheng et al. 2015). Globally and in South Africa, older people often play prominent roles in taking care of their grandchildren, allowing parents to go to work without having to pay for childcare (Pulgaron et al. 2016). This important role could be impacted greatly by reduced functionality when awaiting TKR surgery.

We also found that patients with lower socioeconomic status waited longer to have surgery. About one-third of the patients in the lower socio-economic group included pensioners, and this may partially explain this finding. However, the category also consists of patients who are unemployed or receive a disability grant. Potentially, these patients are not contactable or do not have money for transport to access the service when it is their turn. It is of concern that the socio-economic vulnerable group is waiting longer for care, widening the inequity gap. In addition, we found that almost half of the economically active age group reported being unemployed because of their knee osteoarthritis. The public healthcare system primarily serves economically disadvantaged patients with lower education levels, often engaged in manual labour, and finding alternative employment may not be a viable option. Rehabilitation programmes such as the Good Life with osteoarthritis Denmark (GLA:D) (Skou & Roos 2017) could empower patients to continue employment for as long as possible. However, rehabilitation programmes for people with non-communicable diseases in South Africa are barred by the poor referral rates from physicians as well as the shortage of rehabilitation personnel in the public health care sector ultimately affecting equitable access to care and health programmes (Louw et al. 2023).

We also found that the sociodemographic profile of the sample is comparable to published literature (Cram et al. 2018; Devasenapathy et al. 2020; Scott et al. 2019; Shichman et al. 2023). The majority of patients on this waiting list for TKR surgery were female older than 65 years (mean age 67.5 years). Compared to other developing countries, patients waiting for and receiving TKR surgery are typically two thirds female and has a slightly lower mean age except for China, who has a mean age of 68 years (Devasenapathy et al. 2020; Han et al. 2021). In contrast, developed countries report a slightly higher mean age (67–72 years) from patients receiving TKR except the United States (US), which showed a decrease in the mean age of patients over the previous decade (Cram et al. 2018; Felix et al. 2019). Developed countries, historically characterised by ageing populations, have set a precedent, and now developing countries are also experiencing rapid growth in the ageing population (Hayward & Malay 2018). Projected incidence rates of TKR procedures in the US and Germany are showing that an increase in demand for this procedure will continue, with ageing patients seeking improved mobility and quality of life (Shichman et al. 2023; Yahaya et al. 2021). In South Africa, the increase and ageing of our population could lead to an increase in demand for TKR, and therefore an increase in demand on the rehabilitative support required while awaiting surgery and thereafter. Most of the participants (70%) had a low level of education, only having completed primary school, and although we did not extract data on race, the majority of people accessing public services are from a previously disadvantaged background (Abrahams, Thani & Kahn 2022). Therefore, the low level of education could be expected as during the apartheid regime, individuals from previously disadvantaged backgrounds were discouraged from becoming educated. People with lower levels of education have been found to have worse pre-operative function and pain as well as worse post-surgical outcomes (Luong et al. 2012). Within our context, it is therefore important to develop and implement educational strategies that will facilitate better understanding among patients of the pre- and post-surgical process to promote equitable outcomes (Cheng et al. 2015).

### Clinical implications

We would like to propose that health equity factors such as socioeconomic status, age and other patient characteristics such as life roles and employability should be taken into consideration when screening patients for TKR waiting lists. Based on clinical criteria, younger patients may be better candidates for surgery, but rehabilitation programmes could be better suited to improve their function, allowing them to continue to partake in gainful employment and delay their need for surgery. In some instances, it might also put off their need for surgery altogether, reducing the burden on the tertiary health care and allowing them to prioritise older patients who do not return to function after surgery as quickly as younger people but could regain function and reduce their health risk profile. A more holistic screening (which still includes the current screening tool) and referral for rehabilitation could improve equitable and realistic access to TKR by reducing waiting lists and enable easier access to elective surgery.

### Key strengths and limitations

Our study was conducted during the height of COVID-19 pandemic, which influenced the number of patients added to the waiting list or receiving surgery during the time of data collection; however, a good sample size was reached. Some patients that are on the waiting list reported that their phone numbers changed or has been stolen or that they have poor telecommunication reception where they live, which essentially made them uncontactable. Our study represents a snapshot of the characteristics of patients on the list in urban settings as no patients from more remote and rural areas of the catchment area for the hospital participated in the study because of several reasons. Several years ago, the waiting list was moved from a manual system to an electronic system that affected some of the patients’ contact details, years waited count or documentation. We conducted the study at a tertiary institution where orthopaedic surgeons are in training, and this factor could also have contributed to older patients being regarded to have higher risk profiles in general and less complicated cases are often selected for surgical training to gain experience compared to hospitals with qualified surgeons. However, these practices could have resulted in a reduction in equity to surgical access for older and poorer patients. The electronic system used by the hospital for recordkeeping is scanned from handwritten notes, which often takes weeks to be uploaded, may contain displaced patient notes (stored in the wrong patient folder) or not scanned at all. Not all physician reports are equally completed, and discrepancies have been noted in the thoroughness of patient history taken.

## Conclusion

The aim of our study was to: (1) describe the health equity profile and quality of life of patients awaiting TKR at a single academic hospital in South Africa; and (2) to describe the association between these health equity factors and the waiting time. The key findings were that one in three patients waited 5 years or longer for surgery and not everyone has equitable access to TKR surgery such as elderly patients (> 70 years) and patients from lower socio-economic backgrounds. The current screening protocol for TKR surgery in the public health care sector does not encourage equitable access to surgery. A more holistic screening approach alongside selective surgical prioritisation and rehabilitation could reduce the waiting list and facilitate equitable access to care.
